# Microarray Technology for Major Chemical Contaminants Analysis in Food: Current Status and Prospects

**DOI:** 10.3390/s120709234

**Published:** 2012-07-04

**Authors:** Zhaowei Zhang, Peiwu Li, Xiaofeng Hu, Qi Zhang, Xiaoxia Ding, Wen Zhang

**Affiliations:** 1 Oil Crops Research Institute of the Chinese Academy of Agricultural Sciences, Wuhan 430062, China; E-Mails: zwzhang@whu.edu.cn (Z.Z.); hxf5646@gmail.com (X.H.); zhangqi521x@126.com (Q.Z.); dingdin2355@sina.com (X.D.); zhangwen@oilcrops.cn (W.Z.); 2 Key Laboratory of Biology and Genetic Improvement of Oil Crops, Ministry of Agriculture, Wuhan 430062, China; 3 Key Laboratory of Detection for Mycotoxins, Ministry of Agriculture, Wuhan 430062, China; 4 Laboratory of Risk Assessment for Oilseeds Products, Ministry of Agriculture, Wuhan 430062, China; 5 Quality Inspection and Test Center for Oilseeds Products, Ministry of Agriculture, Wuhan 430062, China

**Keywords:** microarray, chemical contaminants, food safety, mycotoxins, biotoxins, pesticide residues, pharmaceutical residues, review

## Abstract

Chemical contaminants in food have caused serious health issues in both humans and animals. Microarray technology is an advanced technique suitable for the analysis of chemical contaminates. In particular, immuno-microarray approach is one of the most promising methods for chemical contaminants analysis. The use of microarrays for the analysis of chemical contaminants is the subject of this review. Fabrication strategies and detection methods for chemical contaminants are discussed in detail. Application to the analysis of mycotoxins, biotoxins, pesticide residues, and pharmaceutical residues is also described. Finally, future challenges and opportunities are discussed.

## 1. Introduction

Monitoring chemical contaminants in food is important because such contaminants are closely related to human life and animal health, as well as international trade of agricultural products and food commodities [1,2]. Major chemical contaminants include mycotoxins, biotoxins, pesticide residues, and pharmaceutical residues [3–9]. Mycotoxins are worldwide contaminants of foods and feed and can cause health problems and economic losses [10]. Biotoxins are toxic substances produced by and derived from plants and animals [11]. Pesticides are substances or mixture of substances intended for preventing, destroying, repelling or mitigating any pest and weeds. Their residues can cause serious acute and delayed health effects among exposed population groups [12]. Recently, pharmaceutical residues have become an increasingly prominent problem due to the abuse of antibiotics. They can be transferred from the food-producing animals to human beings and diminish the effectiveness of antibiotic treatments for common infections [13]. There have been strict maximum residue limits (MRLs) for many chemical contaminants in many kinds of food, feed, and food-related issues are required by the European Union (EU) and most countries. Legally, MRLs strictly define the maximum permitted concentrations of the residues in or on foodstuffs for humans or animals. The limit of detection (LOD) based on diversified technologies should meet the MRLs.

Different strategies for rapid and on-site assays are being developed to deal with the growing concerns related to chemical contamination. Although high-performance liquid chromatography (HPLC) or gas chromatography (GC) coupled to mass spectrometry (MS) have been successfully applied to monitor chemical contaminants in food and feed [14], microarray-based analytical systems are attractive alternatives due to their high throughputs, high density, high sensitivity, enhanced reproducibility, low sample consumption, reduced analytical time, and ease of automation [15]. The microarray principle relies on specific biomolecular recognition on a certain well-defined heterogeneous substrate. Using microprinting, microspotting, or microstructuring, each probe molecule is patterned on a chosen support to form a highly ordered matrix. The target analytes from samples can be recognized and identified either semi-quantitatively or quantitatively. For recognition of target molecules on microarrays, antibody molecules are most commonly used, providing the specificity and sensitivity to meet the requires of the low EU MRLs. Recently, synthetic molecular recognition elements have been designed and fabricated as affinity materials and applied to the analysis of chemical contaminants. These materials include nanomaterials and membrane structures and can use molecular imprinted polymers, aptamers, phage display peptides, binding proteins, and synthetic peptides as well as metal oxides.

In-depth reviews have been published on DNA microarrays [16–20] and protein microarrays [21–23]. This review, covering selected literatures from 2007 to March 2012, emphasizes their application to the detection of chemical contaminants in foodstuffs.

## 2. Fabrication Method

### 2.1. Solid Support Materials

A successful microarray depends mainly on the design and functionalization of “smart surfaces” to immobilize functional DNA, antigens, or antibodies. There are three major solid support materials used to fabricate microarrays as follows [24]: two-dimensional (2D) support materials include glass, silicon and gold; for three-dimensional (3D) porous materials, macroporous silicon, polyacrylamide, chitosan, hydrogel and agarose gels are utilized [25]. Polymer materials such as polydimethylsiloxane (PDMS) comprise the third type of solid support materials [26,27]. Silicon or gold supports can be modified electrochemically. Glass, the most widely used solid surface support, is resistant to chemical agents and has a stable surface character and reduced autofluorescence. Polymeric support materials are attractive because of the wide range of compositions that are available and their ease of use.

### 2.2. Fabrication of Microarrays

Microarrays allow covalent coupling of numerous probes on solid supports. The immobilization of antibodies and other biomolecules, which are effective against chemical contaminants, on transducers plays a key role in microarray fabrication. Various strategies for linking biomolecules to solid supports include coupling thiols to gold surfaces, acrylamides to silanized surfaces, and amines to aldehyde-treated surfaces [28,29]. Specific coating methods can create a uniform surface for the immobilization of DNA or protein without changing the natural conformation and activity of the biomolecules and can also prevent nonspecific biomolecule adsorption that decreases the analytical sensitivity. There are several spotting methods used for microarray fabrication, e.g., contact printing, microcontact printing, noncontact printing, microfluidics, continuous flow microspotting, and photolithography. After robotically spotting with high-density probes on solid supports, microarrays can identify selected targets. Spotting of probes can use both physical attachment and covalent binding approaches [30]. The physical attachment method facilitates simple fabrication processes. However, attached probes are liable to detach reversibly under high-salt or high-temperature conditions, which reduces hybridization efficiency. Alternatively, covalent bonding methods ensure strong and specific immobilization on solid supports. Thus, it is the preferred technique for surface functionalization.

Microfluidics is a promising approach that can be used to functionalize a well-defined solid support with a specifically addressable recognition zone. A continuous flow microspotter is used to fabricate the microarray sensor chip. In contrast to contact spotting, this instrument yields a microarray having uniform morphology on the spot, without crosstalk. The commercially-available deoxynivalenol-ovalbumin (DON-OVA) and zearalenone-ovalbumin (ZEA-OVA) toxin-protein conjugates have been covalently attached to the surface and coupled via the amine groups of the OVA to a gold substrate coated with preactivated carboxylated polymeric hydrogel. Such a coupling provides a stable array with covalently attached toxin-protein conjugates on the surface [31]. Moreover, multiplexed detection has been demonstrated with a tethered bilayer membrane array built in parallel microchannels. These channels allow multiple measurements to be carried out simultaneously with high precision [32].

The ion milling process can also be used to make a patterned microarray on a spin-valve film with a layer sequence similar to that of hard disk drive read heads. An 8 × 8 microarray was passivated with a tri-layer oxide (SiO_2_ 10 nm/Si_3_O_4_ 10 nm/SiO_2_ 10 nm) that was used for mycotoxin analysis [29]. Another method, screen-printed electrode technology, is gaining interest for the fabrication of microelectrodes [33]. The use of screen-printing, electrochemical, and sonochemical methods has allowed the production of microelectrode arrays for the determination of chlorine in water [34].

Electrochemical detection of chemical contaminants, for which the fabrication of microelectrodes is key, is also possible. Gold cell-on-a-chip microelectrodes, including on-chip reference and counter electrodes, were fabricated via standard deposition, etching, and lithographic techniques used in microfabrication technology [28]. After functionalization with a silicon dioxide layer via plasma-enhanced chemical vapor deposition on a silicon wafer, a patterned photoresist was used to etch the exposed oxide layer. For the fabrication of the metal electrodes, gold was deposited by evaporation of Ti/Pt/Au multilayers (30:50:250 nm). After passivation with a Si_3_N_4_ layer, a recessed microelectrode array (500 nm recess depth) was obtained using a photolithographic etch process (Pt-EKC solvent). To demonstrate the availability of the microelectrode, the characteristic steady-state cyclic voltammograms (CVs) for microelectrodes were recorded at 5 mV/s in 1 mM ferrocene carboxylic acid in phosphate buffered saline. This indicated that there was sufficient interelectrode spacing to achieve independent diffusion profiles for each element of the array. Measured currents agreed with those predicted by established models for diffusion-controlled currents with microelectrodes.

To estimate the efficiency of surface functionalization after probes assembly, various evaluation methods have been used to analyze the topography of surfaces. These include consist of scanning probe microscopy (SEM), atomic force microscopy, infrared microspectroscopy, 2D fractal dimension analysis, perimeter-area relationship and power spectrum density algorithm [35,36].

## 3. Detection Strategies

Detection strategies can be divided into two categories, *i.e.*, labeling and label-free. Both strategies must consider several key detection principles, e.g., limit of detection (LOD), sensitivity, dynamic range, multiplexing and high throughput capability, resolution and specificity.

### 3.1. Labeling Detection

Labeling detection methods are to use probes based on fluorescent dyes. Both fluorescence and chemiluminescence (CL) detection signals arise from probe molecules conjugated to DNA or protein targets. Fluorescence scanning is performed to record the signal by using, for example, Cy5-labeled streptavidin [32]. During the scanning process, fluorescence signal intensities are measured. The relative fluorescence intensity of each individual array feature is quantified by relating the measured fluorescence intensity to the on-chip fluorescence calibration intensity.

Various micro- or nano-scale materials can be employed as fluorescence tags. Three bioterrorism toxins, including ricin, cholera toxin (CT), and Staphylococcal Enterotoxin B (SEB) were simultaneously detected using the inorganic fluorescent dye RuBpy that had been encapsulated in silica nanoparticles (NPs) [37]. Quantum dots (QDs) were used as the probe for multiplexed analysis of pesticides due to their narrow, highly specific, stable emission spectra [38]. Light scattering and fluorescence emission coupled radiometry using QDs as the label were used to analyze pharmaceutical residues [39].

CL can produce a fluorescence effect in some chemical reactions. This behavior can reduce background noise and thereby improve analytical sensitivity. Using this approach, Sauceda-Friebe *et al.* [40] reported a very low limit of 7 μg/kg for ochratoxin A (OTA) using a regenerable microarray, which was lower than the EU MRL of 10.0 μg/kg.

### 3.2. Label-Free Detection

Although the labeling detection method has several advantages, it suffers from high labor and time costs, relatively low throughput, and low sensitivity because of the reduced natural activities of biomolecules. Label-free detection techniques can analyze targets using mass, dielectric, or optical properties.

#### 3.2.1. Electrochemical Detection

Both direct electrochemistry of target molecules and indirect electrochemistry based on molecular recognition have been extensively studied using electrochemical microarrays [41]. A detection strategy based on electrochemical detection has been used for either single or multi-analyte assays [28,33]. Enhanced mass-transport has been reported for a microelectrode array. Hemispherical diffusion layers are formed at such microelectrodes, and a much faster diffusion of electroactive substances occurs because of the multidimensional nature of this process. This behavior results in sigmoidal (or steady-state) CVs. The fabrication of microelectrodes on a substrate and the immobilization of various antibodies or other molecules for recognition on these microelectrodes have the advantages of reduced transduction rate, enhanced sensitivity, and increased responding signal per unit electrode surface area (greater current density, higher signal-to-noise ratio). Advances in photolithography techniques have enabled the patterning of microelectrodes of various designs with excellent reproducibility at microscale dimensions on suitable wafers. Electrochemical analysis with microarrays benefits from excellent sensitivity, selectivity, versatility, and simplicity. The enzyme labeling methodology amplifies electrochemical signals; thus, small amounts of enzyme-generated product can be amplified and then detected by the electroanalytical technique. As a result, there is growing interest in this approach for environmental [42], food [43,44], and clinical science [45] applications.

#### 3.2.2. Surface Plasmon Resonance

Surface plasmon resonance (SPR) is a new label-free technique that is normally used to investigate molecular interactions. A continuous change of refractive index can be recorded by SPR on a biorecognition layer of the microarray surface. It is readily adapted to various microarray-based sensors [46,47] to provide quantitative or qualitative information on the chemical contaminants. The advantages of this technique include the ability to monitor the binding interactions of immunoreagents and to analyze target molecules without any expensive and time-consuming labeling procedure [48]. However, many low molecular weight chemical contaminants are too small to induce significant changes in refractive index upon binding to the microarray surface. One solution to enhance SPR sensitivity is the bioconjugation of target molecules with a high molecular weight carrier such as a bovine serum albumin or OVA. Diverse detection formats can be applied for designing SPR microarrays, among which the competitive inhibition format is probably the most used and most robust for the detection of small organic molecules such as chemical contaminants, especially multiplexed mycotoxins [31]. The layer-by-layer method for detecting signal amplification is another useful technique to discriminate target signals from background noise. For example, a simple alternative to SPR imaging (SPRi) analysis of CT relies on the conjugation of avidin and biotinylated anti-avidin, an amplification strategy that was initially proposed for immunosensing [32].

## 4. Microarray Based Analysis of Major Chemical Contaminants

Mycotoxins, biotoxins, pesticide residues, and pharmaceutical residues are the major chemical contaminants found in food. Microarray can realize can realize on-site analysis and monitor real-time data, leading to an enormous cost-saving to risk assessment on food.

### 4.1. Mycotoxins

Mycotoxins, toxic secondary metabolites produced by filamentous fungi, have received considerable attention [49] because of their health risks to both humans and animals. Microarray technologies have played an important role in mycotoxin analysis because of their speed, sensitivity, and cost-saving [41].

Electrochemical immunoassay-based microarrays have been successfully used for mycotoxin analysis because of their sensitivity, selectivity, versatility, and simplicity. Microelectrodes have greater sensitivity because of enhanced mass-transport on microscale electrodes. The most widely used method for mycotoxin recognition on a microarray depends on the antibody molecule, which can offer the high specificity and sensitivity required for low-level mycotoxin detection. A microelectrode immunosensor-based microarray for aflatoxin M1 (AFM1) has been investigated [28]. The microelectrode arrays consisted of 35 microsquare electrodes with dimensions of 20 μm × 20 μm and an edge-to-edge spacing of 200 μm. The on-chip reference and counter electrodes were fabricated via standard photolithographic methods. Cyclic voltammetry was used to determine the characteristics of the microelectrode arrays and the behavior of the on-chip electrodes. To analyze for AFM1 directly in milk samples, antibodies against AFM1 were immobilized by cross-linking with 1,4-phenylene diisothiocyanate on the surface of the microarray, which was pre-functionalized with silanization reagent. Without matrix interference, a cELISA assay format was conducted on the microarray electrode surface using 3,3,5′,5′-tetramethylbenzidine dihydrochloride/H_2_O_2_ with horseradish peroxidase as the enzyme label in an electrochemical detection mode. The LOD for AFM1 in milk was 8 ng/L with a dynamic detection range of 10–100 ng/L, which was lower than the current EU legislative MRL of 50 ng/L. Compared with other recent reports on the analysis of AFM1 using electrochemical detection (e.g., an electrochemical immunosensor based on magnetic NPs coated with antibody and screen-printed carbon electrodes (LOD: 50 ng/L) reported by Paniel *et al.* [50] and an impedimetric biosensor based on a DNA probe and gold NPs (LOD: 39 ng/L) reported by Dinckaya *et al.* [51], this method demonstrated a lower LOD and comparable dynamic detection range. This highlighted the advantages of using microarray technology.

Radi *et al.* [33] used a screen-printed gold electrode for the sensitive detection of OTA. After modification via electrochemical and chemical reactions, amine-modified microelectrodes can be covalently bound with antibodies against OTA. A competitive immunoassay was further demonstrated via a competition between OTA and a horseradish peroxidase-labeled OTA (OTA-HRP) for the immobilized antibodies. The activity of the bound OTA-HRP was electrochemically analyzed by chronoamperometry using 3,3′,5,5′-tetramethylbenzidine as the substrate. The measured LOD of 12 ng/mL and a dynamic range up to 60 ng/mL for OTA analysis was comparable to the EU MRL of 10 ppb. Mak *et al.* [29] advanced multiplex mycotoxin analysis via the integration of the typical sandwich immunoassay into a modified magnetic nanotag (MNT) detection platform. MNT was adapted to detect target molecules that are smaller in size (<300 Da) and insoluble. Real-time detection was realized via the addition of MNTs onto the spin-valve sensor surface that had been previously immobilized with capture antibodies against mycotoxins and secondary antibodies. Capture antibodies were immobilized on different parts of the GMR sensor surface. After the addition of the MNT into the sandwich immunoassay system, the signal related to the streptavidin-avidin binding kinetics was recorded in real time. The results demonstrated high specificity, high capability of multiplexed analysis, and reduced LODs of 50 pg/mL for AFB1, 0.05 ng/mL for ZEA, and 333 pg/mL for HT-2. These LODs met the EU MRLs (AFB1: 5–12 μg/kg; ZEA: 20–350 μg/kg).

More recently, SPR has been explored as a rapid label-free screening method for the detection of food contaminants, in particular mycotoxins. SPRi allows multiplex screening of tens of different biointeractions using a microarray of sensing spots. The maximum number of mycotoxins detected using an SPR microarray is limited to 4 [52]. Dorokhin *et al.* [31] reported an elaborate SPRi-based microarray platform used for the detection of deoxynivalenol (DON) and zearalenone (ZEA) via a competitive inhibition immunoassay format. A continuous flow microspotter device was used in the fabrication. LODs of 84 and 68 μg/kg for DON and 64 and 40 μg/kg for ZEA were found for maize and wheat samples, respectively, suggesting that this microarray method could satisfy EU MRLs (ZEA: 350 μg/kg for maize and 100 μg/kg for wheat; DON: 1,750 μg/kg for both maize and wheat). The results using a single microarray chip were in good agreement with LC-MS/MS data. This method easily met the EU regulatory limits for mycotoxin contaminants in food and feed samples. Furthermore, the results suggested that the SPRi microarray chip platform holds promise for the development of a rapid multiplex screening method for up to 40 different mycotoxins.

**Figure 1.**
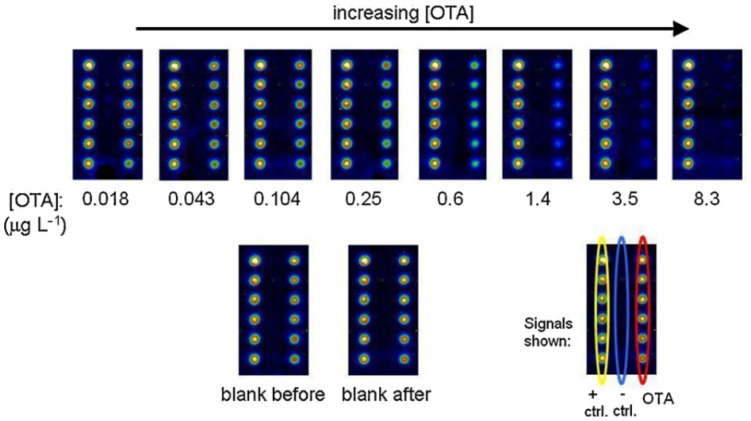
Images of the biochip in sequential experiments with increasing OTA concentration including blanks measured before and after calibration. (Reprinted from [40] © 2011 Elsevier B.V.).

A regenerable, reusable microarray is of growing interest because of its improved reusability and environmentally-friendly characteristics. Sauceda-Friebe *et al.* [40] developed a regenerable microarray for screening OTA in green coffee extract on a fully-automated flow-through device with a CL readout (Figure 1). After synthesis of a water-soluble peptide-OTA conjugate and peptide-biotin conjugate for covalent immobilization on a glass support by contact spotting, the microarray was used in an indirect competitive immunoassay format with flow-through reagent addition and CL detection. On-line mixing and sequential pumping of solutions over the microarray surface modified with the analytes of interest were carried out. Over 20 assay-regeneration cycles of the microarray surface were completed by repeated covalent conjugate immobilizations. The results indicated a reduced assay time of 12 min and a limit of quantitation (LOQ) of OTA in green coffee extract of 0.3 μg/L, which corresponds to 7 μg/kg. The LOQ is comparable to the EU MRL of 10.0 μg/kg. Before starting a new assay cycle, a rebinding procedure was performed with a regeneration solution having acidic pH and high ionic strength. The solution was pumped through the flow cell to remove the streptavidin-HRP. There was a continuous reduction in signal intensity over several regeneration cycles because of the imperfect disruption of the biotin-streptavidin complex, leading only to its partial removal from the chip surface.

### 4.2. Biotoxins

It is accepted that biotoxins, produced by bacteria, plants, or animals, are a major concern in food safety because of their extremely high toxicities and their potential use as biological warfare agents. Biotoxin analysis based on microarrays has been successfully developed by several groups [53–57] and is still an active research area.

Non-specific protein adsorption can affect the analysis of chemical contaminants as a result of hydrogen bonding, charge interactions, or non-polar interactions. For the detection of enterotoxin B (EB) using a microarray, three polyethylene glycol (PEG) derivatives (PEG-methacrylate, PEG-diacrylate, and PEG-dimethacrylate) were grafted onto galactose-based polyacrylate hydrogels to reduce non-specific protein adsorption [25]. A PDMS template containing microchannels was used to conduct sandwich immunoassays to detect SEB. In these hydrogel microarrays, fluorescence laser scanning confocal microscopy of a Cy3-labeled tracer antibody specific for SEB and ricin provided both qualitative and quantitative data for the detection sensitivity and the reduction in non-specific binding as a result of PEG incorporation. In this way, a LOD for SEB of 1 ng/mL was found, showing a ten-fold decrease in the non-specific binding and a six-fold increase in specific binding of SEB. For the application of homogeneous solution-phase-based multiplexed assays for SEB analysis, microbeads with specific fluorescent signatures have been used for the pre-immobilization of immunoreagents (antibodies). Mulvaney *et al.* [58] demonstrated a microarray-based method for SEB detection with only two reagent mixtures and three assay steps with a reduced time of 10 min by using a semi-homogenous implementation of a fluidic force discrimination assay. After capture by an appropriate intermediate receptor, such as secondary antibodies, the beads were specifically captured onto a microarray support. Results showed a 1,000-fold improvement in assay sensitivity down to attomolar concentrations, *i.e.*, an LOD for SEB of 35 aM (1 fg/mL). A 100-fold improvement in reaction rate by delivering target-laden microbeads to the microarray was reported as well.

A label-free method using SPRi, as presented by Taylor and coworkers [32], was employed in a microfabrication approach to generate well-defined, addressable, and regenerable lipid membrane microarrays in PDMS microchips for CT analysis. The nonionic surfactant Triton X-100 could regenerate the surface of the microarray more than three times, suggesting that the microarray interface was highly reproducible. The results were linear over the range of 25 to 175 μg/mL. The LOD for CT was 260 nM. Lian *et al.* [37] used a novel fluorescent NP with a sensitive antibody microarray assay system to detect bioterrorism agents comprising ricin, CT, and SEB. A sandwich format was employed, which consisted of capture antibodies, target toxins, biotinylated detection antibodies, and avidin-conjugated NPs. Polyclonal antibodies (pAbs) were better than monoclonal antibodies (mAbs) at capturing toxins on microarray supports. When used in the detection of toxins spiked in milk, apple cider, and blood samples, the LOD of ricin in spiked apple cider or milk was 1 ng/mL, while CT and SEB could be detected at 100 pg/mL in spiked apple cider or milk. Rubina *et al.* [59] reported the simultaneous analysis of seven staphylococcal enterotoxins (SEs), A, B, C1, D, E, G, and I, in a single sample by using hydrogel-based microarrays. After the expression and purification of recombinant toxins, the selected mAbs had high affinity toward their targets and no cross-reactivity with unrelated enterotoxins. A high sensitivity ranging from 0.1 to 0.5 ng/mL was found for SEs. When used to detect spiked food samples, the excellent sensitivity, specificity, and reproducibility of microarrays fully satisfied the EU limit for SEs. Moreover, the rapid assay of SEs on a metal-coated microarray could reduce the time of the simultaneous quantitative sandwich immunoassay of seven enterotoxins from 17 to 2 h without loss of sensitivity, when compared with the assay on regular glass supports. The CombiMatrix antibody microarray, based on the generation and transduction of electrochemical signals following antigen binding to surface antibodies, was introduced by Wojciechowski *et al.* [44]. They detected SEB along with inactivated *Y. pestis*. Using HRP to oxidize the substrate, the reported LODs were 5 pg/mL and 106 CFU/mL, respectively. A super avidin-biotin system was developed as a viable and effective means to enhance the sensitivity. SPRi was also employed by Linman *et al.* [60] as a label-free detection approach for CT on a gold-coated etched glass microarray; an LOD of 5 nmol/L was obtained. The etching of the glass support significantly improved the performance, in particular the image contrast and sensitivity. This was because the resonance condition changed, *i.e.*, the resonance angle between the etched wells and the surrounding area, leading to isolation of the array spot resonance with a significant reduction of the background signal. Furthermore, the microarray surface could be regenerated by Triton X-100 for repeated cycles of membrane formation, protein binding, and biomolecular removal. Finite-difference time-domain simulations showed that microarrays with large spots and minimal spot-to-spot spacings could provide ideal differential resonance conditions and that the SPR electric field intensity for the etched well structure was three times higher than for standard planar gold chips. Asanov *et al.* [61] used a total internal reflection fluorescence (TIRF) microarray in simultaneous bioassays of DNA and protein to obtain a better signal-to-background ratio. Analysis speed and simplicity were improved with the TIRF microarray, and it could record an entire course of association and dissociation kinetics between target DNA and protein molecules. A TIRF microarray system can be mounted on microscopes or interfaced directly with charge-coupled device (CCD) cameras equipped with a single objective. In this manner, the molecular markers from *Bacillus anthracis*, the pathogen responsible for anthrax, were successfully detected.

A microarray-based simultaneous detection method was also employed in the multiplexed analysis of bacterial and plant toxins by using the specificity of covalently immobilized capture probes, as described by Weingart *et al.* [62]. A microstructured polymer slide was used both as the support of the printed microarrays and as the incubation chamber. Fluorescence image using Cy5-coupled streptavidin as a probe provided quantitative determination of the Botulinum Neurotoxin Type A (BoNT/A), SEB, and the plant toxin ricin. It displayed good performance, including an LOD of 0.5–1 ng/mL in buffer or in raw milk.

### 4.3. Pesticide Residues

Pesticides are substances or mixture of substances that prevent, destroy, repel, or mitigate any pest. Pesticide residues can be retained in the upper soil and groundwater and can be transferred by water into the food chain.

**Figure 2.**
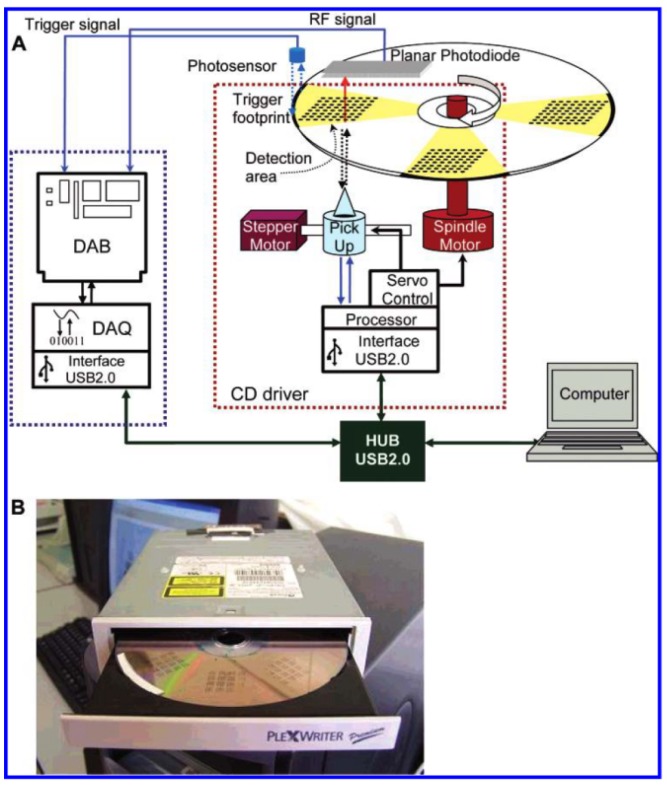
(**A**) Schematic representation of the detection system. In the blue dashed outline: DAB, DAQ and mMachine interface, and in the red dashed outline: CD drive system, power section and processor; (**B**) Picture of the CD player used in this work (modified from [63] © 2007 American Chemical Society).

To monitor water pollution, Davis *et al.* [34] reported a simple-to-use, disposable microarray suitable for the rapid assay of pollutants in aqueous media. Their microarray was fabricated by a sonochemical microelectrode fabrication technique. The incorporation of enzyme-containing conductive polymers could dramatically reduce the LOD at concentrations as low as 10^−17^ M when detecting organophosphate pesticides. This satisfies EU MRLs that range from 0.05 to 0.1 μg/kg. High-density competitive indirect microimmunoassays were developed by Morais *et al.* [63] on low-reflectivity compact disc (CD)-based microarrays by direct absorption of immunoreagents onto the polycarbonate surface (Figure 2).

Gold- or enzyme-labeled immunoglobulins were used as tracers to display the immunoreaction. The optimal CD reflected 30% and transmitted 70% of the CD reader laser beam (λ = 780 nm); the reflected light was used to scan the disc track, while the transmitted light was detected via a planar photodiode that was integrated on the CD drive. For quantitative detection, the variation of optical transmission of the light source that originated from the immunoreaction products was recorded. Pesticides were identified in a 60-min assay time, with LODs ranging from 0.02 to 0.62 μg/L for 2,4,5-trichlorophenoxypropionic acid, chlorpyriphos, and metolachlor. These LOD values suggest that this microarray could meet the MRLs of the EU.

**Figure 3.**
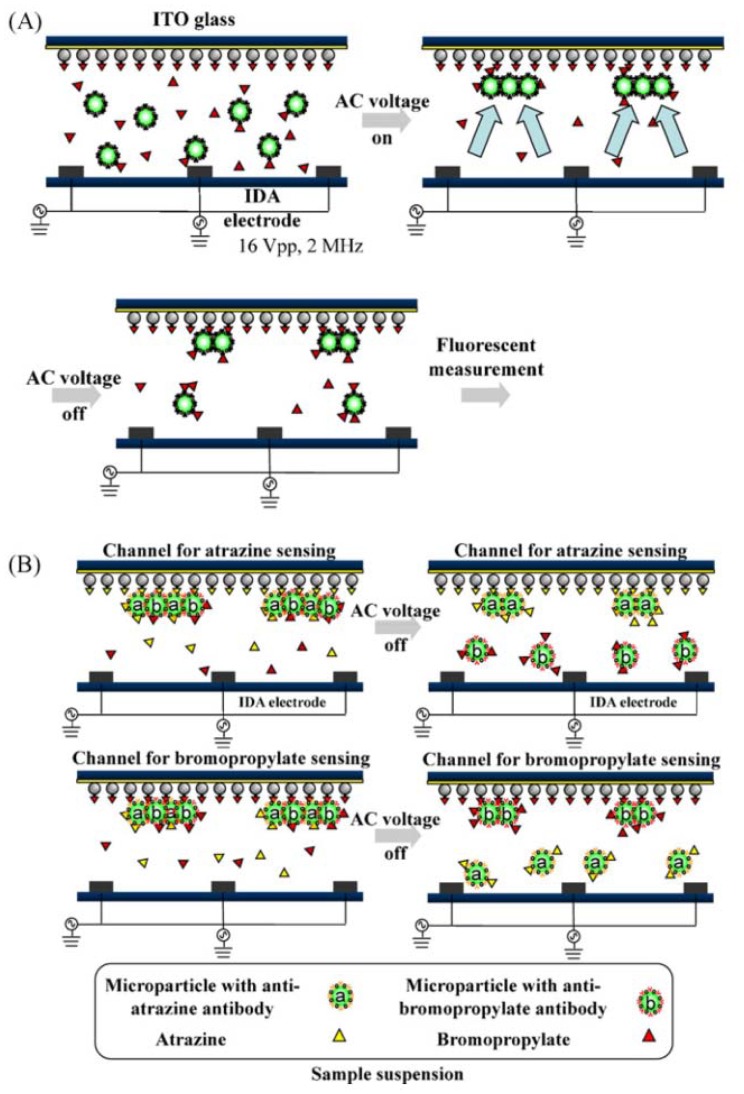
Schematic representation of accelerated immunosensing based on the n-DEP device. (**A**) Single analyte sensing system and (**B**) multi-analyte system usingthe n-DEP device with two channels modiӿed with different competitors. Arrows in the channel represent the direction of the manipulation of microparticles with the n-DEP force. (Reprinted from [64] © 2010 Elsevier B.V.).

Nichkova *et al.* [65] established the use of QDs as a probe for a multiplexed immunoassay of 3-phenoxybenzoic acid and atrazine mercapturate, which presented as pyrethroid and herbicide atrazine, respectively. Coating antigens were fabricated by microcontact printing and placed in line patterns on the glass support. The multiplexed competitive immunoassays were characterized by fluorescence microscopy and SEM. Ramon-Azcon [64] used a rapid and separation-free immunosensing system to identify two pesticides that are widely used in food samples, atrazine and bromopropylate (Figure 3). They were identified in less than 5 min by competitive immunosensing based on rapid manipulation of microparticles with negative dielectrophoresis (n-DEP), in which a force was exerted on a dielectric particle when it was subjected to a non-uniform negative electric field.

The results showed LODs of 4 and 1.5 μg/L for atrazine and bromopropylate, respectively, without separation procedures. These LODs were sufficient to meet international regulations regarding pesticide residues in food samples (MRL in the EU for atrazine and bromopropylate: 0.1 μg/kg). Antibody-modified fluorescence microparticles were injected into the interdigitated microarray electrode via n-DEP. In the same group, another sensitive and spatially multiplexed microarray based on n-DEP manipulation of DNA-encoded particles was developed for analysis of atrazine and bromopropylate [66]. These particles could be accumulated by n-DEP for competitive immunoassay on microparticles, then captured via hybridization among single-stranded DNAs (ssDNA). A mixture of two types of microparticles pre-functionalized with antibodies specific to atrazine and bromopropylate was introduced into the microarray platform, in which electrodes and two ssDNAs for both pesticides were previously conducted. A rapid accumulation on the encoded surface areas was achieved with n-DEP. On this detecting platform, atrazine and bromopropylate were simultaneously detected with LODs of 0.2 μg/L; this level covered the MRL in food samples established by the EU and the Japan Ministry of Health, Labor and Welfare (MHLW).

### 4.4. Pharmaceutical Residues

Pharmaceutical residues herein refer to residues from veterinary drugs and antibiotics. A number of pharmaceutical compounds used in animal medical care are released into the surroundings and then found in food. Microarray technology has been successfully applied to analyze pharmaceutical residues [67–69].

Qie and coworkers [39] developed Rayleigh light scattering and fluorescence emission coupled with radiometry. By using CdS QDs as labels, aminoglycoside antibiotics were detected in serum samples. After attachment on the surface of CdS QDs via electrostatic interaction in aqueous medium, aminoglycoside antibiotics exhibited strong enhanced light scattering emission at 376 nm and fluorescence quenching of the CdS QDs at 500 nm. The coexistent light scattering and fluorescent emission signals were recorded in order to establish a linear relationship for the analysis of aminoglycoside antibiotics from aminoglycoside antibiotics injection and serum samples. The LOD (3σ) of 58–190 nmol/L showed a satisfactory result compared with 500 ppb for the MRLs in the EU. Adrian *et al.* [70] proposed a typical planar microarray to detect sulfonamide, fluoroquinolone, and β-lactam antibiotics in milk samples based on class-selective bioreceptors by using the combination of two independent ELISA for sulfonamide and fluoroquinolone antibiotics and an enzyme-linked receptor assay for β-lactam antibiotics. On a microarray pre-coated in specific sections with the corresponding haptenized proteins, a mixture of samples with a cocktail containing the bioreagents was introduced in the microplate wells, where a positive response indicated the presence of antibiotics. This multianalyte method provided a single assay for over 25 different antibiotics from the three most important antibiotic families. The detectability in full-fat milk samples achieved the EU MRLs. Morais *et al.* [71] studied an herbicide sample using a sensitive and versatile microarray that involved recordable CDs as target screening surfaces and a standard optical CD/DVD drive as a detector. Atrazine was detected via quantitative immunoanalysis by using enzyme or gold NP-labeled antibodies as tracers. Results showed a LOD for atrazine (0.04 μg/L) below the maximum EU residue limit for drinking water. This improved CD reading depended on the capture of analog signals with the disk drive that were proportional to the darkness of the immunoreaction product.

**Figure 4.**
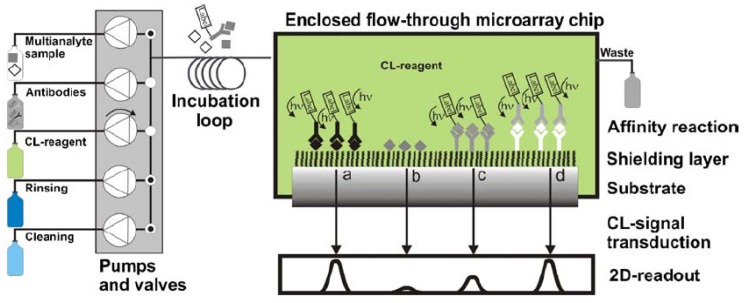
Schematic set-up of an analytical CL flow-through microarray platform for quantifying of analytes with indirect (a and b), direct (c), and sandwich (d) assay formats. (Reprinted from [72] © 2008 Elsevier B.V.).

One major way that pharmaceutical residues can enter the food chain is through waste water. To address this issue, Pastor-Navarro *et al.* [73] reported a microarray method for sulfamethoxazole analysis via a microarray on a CD support, resulting in an LOD of 0.09 ng/mL. On a polycarbonate surface of a low-reflectivity CD, competitive microimmunoassays were conducted by direct adsorption of immunoreagents, followed by nanogold-labeled immunoglobulins and silver staining developer. This CD displayed a high analytical capacity (2,560 spots per disc), with a mean recovery of 87%. Kloth *et al.* [72] presented an automated CL read-out system for reusable flow-through microarrays based on multiplexed immunoassays of antibiotics in milk (Figure 4). The calibration and determination of antibiotics were done automatically. No less than 13 different antibiotics in milk could be automatically detected within 6 min via an indirect competitive immunoassay, in which antibiotics were directly immobilized on epoxylated PEG surfaces. Results of penicillin analysis demonstrated regeneration for 50 measurement cycles per channel [74]. An LOD of 1.1 μg/L was achieved in an assay time of 6 min, meeting the MRLs of 5 μg/kg in the EU.

According to Raz *et al.* [75], four major antibiotic residues in milk, namely, aminoglycosides, sulfonamides, fenicols, and fluoroquinolones, were detected in a competitive format by using an SPRi-based microarray and a label-free and multiplex detection platform. An LOD at the ppb level was found in buffer and in 10× -diluted milk, suggesting that this microarray was sensitive enough for analysis of aminoglycosides (neomycin, gentamicin, kanamycin, and streptomycin), sulfonamides (sulfamethazine), fenicols (chloramphenicol), and fluoroquinolones (enrofloxacin) for milk quality control at the maximum allowable residue levels of the EU. Zhong *et al.* [69] reported the analysis of clenbuterol and sulfamethazine using competitive immunoassay in which artificial antigens were spotted on microarray slides. Results showed that the reduced IC50 values were 39.6 ng/mL for clenbuterol and 48.8 ng/mL for sulfamethazine, compared with 190.7 and 156.7 ng/mL for clenbuterol and sulfamethazine, respectively, by traditional indirect competitive ELISA; recovery was increased by 90%, and the LOD was 0.9 ng/g for clenbuterol, comparable to the MRL of 0.5 μg/kg. Recently, Wutz *et al.* [76] presented a simultaneous determination method for four different antibiotic residues (enrofloxacin, sulfadiazine, sulfamethazine, and streptomycin) in honey by using regenerable antigen microarrays via CL in indirect competitive immunoassay format without a purification or extraction procedure. The surface of a glass support was pretreated with epoxy-activated PEG in order to directly immobilize antibiotic derivatives, while the CL read-out via a CCD camera was used for detection. An effective data evaluation method provided fast and automated processing. Spiking experiments revealed adequate recoveries within the dynamic ranges of the calibration curves of enrofloxacin (86–98%), sulfamethazine (109–151%), sulfadiazine (69–109%), and streptomycin (89–97%).

## 5. Conclusions

There has been an increasing demand for improved analytical methods for chemical contaminants in food. In addition, there are increasing concerns for food safety, growing food trade disputes, and increasingly stringent government regulations. Microarray technology has been used for the past five years for the detection of chemical contaminants in food. A selected literature review has been discussed that includes fabrication strategies, detection methods, and application to four classes of chemical contaminants in food.

Microarray technology offers a powerful strategy for analysis of the chemical contaminants in food. However, to this day, only limited advances have been made using microarrays for the identification of chemical contaminants. There are several reasons for this. First, simplification of fabrication is highly desired because contact or noncontact spotting requires special spotting applicators. Although biochip microarrayers have been fully commercialized, more portable methods such as microcontact printing-based spotting, are preferred. Second, miniaturization of the detection system is beneficial for lower energy consumption and miniaturization of the whole microarray system. Third, multiplexed cross-border analysis would be particularly useful, e.g., simultaneous analysis of both mycotoxins and pesticide residues or even both chemical contaminants and pathogenic bacteria.

There is a trend to combine emerging advanced analytical technologies with microarray chemical contaminants. Microarray technology is a promising tool for the monitoring of food safety and provides a greater understanding of food-borne chemical contaminants.
